# Serum proteins may facilitate the identification of Kawasaki disease and promote in vitro neutrophil infiltration

**DOI:** 10.1038/s41598-020-72695-z

**Published:** 2020-09-24

**Authors:** Sung-Chou Li, Kuo-Wang Tsai, Lien-Hung Huang, Ken-Pen Weng, Kuang-Jen Chien, Yuyu Lin, Chi-Ying Tu, Pei-Hsien Lin

**Affiliations:** 1 Genomics and Proteomics Core Laboratory, Department of Medical Research, Kaohsiung Chang Gung Memorial Hospital, Chang Gung University College of Medicine, Kaohsiung, Taiwan; 2 Department of Research, Taipei Tzu Chi Hospital, Buddhist Tzu Chi Medical Foundation, New Taipei, Taiwan; 3 Department of Neurosurgery, Kaohsiung Chang Gung Memorial Hospital, Chang Gung University College of Medicine, Kaohsiung, Taiwan; 4 Congenital Structural Heart Disease Center, Department of Pediatrics, Kaohsiung Veterans General Hospital, No.386, Dazhong 1st Rd., Zuoying Dist., Kaohsiung, Taiwan; 5 Department of Medicine, National Yang-Ming University, Taipei, Taiwan; 6 Shu-Zen Junior College of Medicine and Management, Kaohsiung, Taiwan

**Keywords:** Immunology, Biomarkers, Diseases, Medical research, Molecular medicine, Pathogenesis, Rheumatology

## Abstract

Kawasaki disease (KD) usually affects the children younger than 5 years of age and subsequently causes coronary artery lesions (CALs) without timely identification and treatment. Developing a robust and fast prediction method may facilitate the timely diagnosis of KD, significantly reducing the risk of CALs in KD patients. The levels of inflammatory serum proteins dramatically vary during the onsets of many immune diseases, including in KD. However, our understanding of their pathogenic roles in KD is behind satisfaction. The purpose of this study was to evaluate candidate diagnostic serum proteins and the potential mechanism in KD using iTRAQ gel-free proteomics. We enrolled subjects and conducted iTRAQ gel-free proteomics to globally screen serum proteins followed by specific validation with ELISA. Further in vitro leukocyte trans-endothelial model was also applied to investigate the pathogenesis roles of inflammatory serum proteins. We identified six KD protein biomarkers, including Protein S100-A8 (S100A8), Protein S100-A9 (S100A9), Protein S100-A12 (S100A12), Peroxiredoxin-2 (PRDX2), Neutrophil defensin 1 (DEFA1) and Alpha-1-acid glycoprotein 1 (ORM1). They enabled us to develop a high-performance KD prediction model with an auROC value of 0.94, facilitating the timely identification of KD. Further assays concluded that recombinant S100A12 protein treatment activated neutrophil surface adhesion molecules responsible for adhesion to endothelial cells. Therefore, S100A12 promoted both freshly clinically isolated neutrophils and neutrophil-like cells to infiltrate through the endothelial layer in vitro. Finally, the antibody against S100A12 may attenuate the infiltration promoted by S100A12. Our result demonstrated that evaluating S100A8, S100A9, S100A12, PRDX2, DEFA1 and ORM1 levels may be a good diagnostic tool of KD. Further in vitro study implied that S100A12 could be a potential therapeutic target for KD.

## Introduction

Kawasaki disease (KD), an acute systemic vasculitis with a predilection for Asian race, occurs mainly in infants and children under 5 years of age^1,2^. KD is liable to be complicated by the development of coronary artery lesions (CALs), which develop in approximately 15–25% of untreated KD children and in approximately 5% of those treated with IVIG therapy^1,2^. Treatment with a single high dose of IVIG is effective in reducing the incidence of CALs^1,2^. However, 8–38% of children will have persistent or recrudescent fever after initial IVIG treatment and are at increased risk for the development of CALs^3–6^. The annual incidence of KD in Taiwan is estimated to be 67.3/100,000 children, which is the third highest in the world after that in Japan and Korea^7^. Delayed diagnosis and treatment of KD (especially atypical KD) may result in a high risk of CALs^2,6^. Recently, the pandemic of COVID-19 has become a serious public health problem, severely threatening people worldwide^8^. Covid-19 infection in children could be associated with KD-like multisystem inflammatory syndrome^9^, which highlighted the need of investigating the association between KD and COVID-19.

The diagnosis of KD depends on the clinical features. This multisystemic vasculitis is characterized by prolonged fever, polymorphous skin rash, nonpurulent conjunctival injection, extremity changes, oral mucosal changes, and cervical lymphadenopathy^2^. However, these clinical features are not objective and may delay the early diagnosis and timely treatment of KD. To overcome this difficulty, many studies have been dedicated to developing KD identification biomarkers to facilitate early identification of KD onset and/or complications^2,10–18^. However, many previous studies relied on dealing with fragile RNA samples. Owing to the characteristic of long-term durability, serum proteins are also investigated for KD identification biomarkers. These serum-related studies were usually initiated by screening for specific serum proteins, which confined the results to a small number of candidate proteins (see “Discussion” for details). As a result, novel serum protein biomarkers were rarely identified. In addition, owing to the lack of a good in vitro cell model for KD function, few of the previous studies further investigated the pathogenesis mechanisms of their identified KD biomarkers.

Isobaric tagging for relative and absolute quantification (iTRAQ) gel-free proteomics has emerged as a powerful tool for discovering candidate protein biomarkers in proteomic studies^19,20^. In this study, we first enrolled healthy controls (HC, healthy subjects without fever), fever controls (FC, subjects with fever but without KD diagnosis) and KD subjects. Then, we applied iTRAQ to globally screen serum samples in FC and KD subjects to detect as many serum proteins as possible. The subsequent ELISA validation identified KD biomarkers which enabled us to develop a high-performance KD prediction model. By using an in vitro cell model applied in a previous study^21^, we also investigated the pathogenic roles of S100A12 in KD. We concluded that S100A12 treatment promoted neutrophil cell infiltration through the endothelial layer in vitro. Such promotion may be attenuated by S100A12 antibody. Therefore, antibody against S100A12 has the potential to serve as an alternative therapy to augment traditional IVIG administration.

## Methods

### Subject enrollment and ethics issue

We enrolled Han Chinese subjects at the department of Pediatrics, Kaohsiung Veterans General Hospital (KVGH), Taiwan. This study was approved by the KVGH institutional ethics board (IRB approval number VGHKS19-CT2-22). All subjects or their guardians signed the informed consent form. All protocols were carried out in accordance with the relevant guideline and regulations. In total, 37 healthy control (HC, without fever), 38 fever control (FC, with fever but not diagnosed as KD) and 93 KD subjects were enrolled in this study. We enrolled healthy control children from our ordinary patient department. These children underwent health checkup and blood drawing for laboratory examinations, such as complete blood count, serum biochemistry, and etc. The remaining blood samples were applied for this study with the approval from their guardians. We enrolled the fever control children with fever (body temperature ≥ 38 °C) for at least 3 days and with evident respiratory tract infections diagnosed, including acute pharyngitis, acute bronchitis, acute tonsillitis, and acute bronchopneumonia. In addition, by using questionnaire and referring to medical records, we excluded the HC and FC subjects with medical history of KD, autoimmune disease, allergic disease, or cardiovascular disease.

No atypical KD subject was enrolled in this study. All KD children underwent two-dimensional echocardiography at the time of diagnosis and again two, four, eight and 12 weeks and six months after treatment, and annually in follow-up. Among the KD patients, 16 subjects had CALs and 14 were IVIG non-responsive. To avoid noises by drugs, we excluded the patients with steroid treatment. All HC and FC subjects were subjected to drawing blood for only one time. Most KD subjects were subjected to drawing blood for three times, namely before IVIG administration, three days after IVIG administration and three weeks after IVIG administration.

### Blood collection

At each run of blood drawing, all subjects donated two tubes of blood (3 ml per tube), one of which was for serum collection and the other for total white blood cell (WBC) collection by red blood cell (RBC) lysis. The collected serum samples were stored at − 80 °C. We used the mirVana miRNA Isolation Kit (Ambion, CA, USA) to extract RNA from total WBC following the manufacturer’s protocols.

### iTRAQ gel-free proteomics to globally screen serum proteins

To globally screen serum proteins, we randomly selected 12 age- and gender-matched FC and 12 KD serum samples. The selected serum samples were first subjected to high-abundance protein depletion with the Pierce Top 12 Abundant Protein Depletion Spin Columns (85165, Thermo). Then, the serum samples of six subjects were evenly pooled, resulting in two pooled FC and two pooled KD samples. The four pooled serum samples were then subjected to sample preparation with the iTRAQ Reagents Multiplex Kit (4352135, Sciex). After passing the standard QC check, the labeled serum samples were analyzed with LC/Q-Exactive Orbitrap MS (Thermo) for 24 h and the generated raw data was analyzed with Proteome Discoverer v2.4 (Thermo) by referring to the MASCOT 2.5 database (Matrix science). As a result, we acquired the relative abundances of detected proteins.

### ELISA to specifically measure the concentrations of serum proteins

Among the detected proteins by iTRAQ, we selected six for ELISA in all serum samples. S100A proteins were selected because their roles in neutrophil infiltration in our previous study^22^. PRDX2, ORM1 and DEFA1 were selected because their variation were consistent between two FC and two KD samples. In addition, the three proteins were related to oxidative stress, inflammation or infection through literature search. We used ELISA to determine the absolute concentrations of serum proteins by referring to the instructions of the ELISA kit manufacturers. The ELISA kits are as follow: S100A8 (CY-8061, MBL, Japan), S100A9 (CY-8062, MBL, Japan), S100A12 (CY-8058V2, MBL, Japan), DEFA1 (ARG82004, Arigo, Taiwan), ORM1 (EG5001-1, AssayPro, U.S) and PRDX2 (KA4801, Abnova, Taiwan).

### Promoter methylation assay and qPCR assay

We conducted promoter methylation assay by mimicking a previous study^23^. The putative promoter region of the S100A12 gene was PCR amplified, followed by being digested with the restriction enzyme HindIII and being cloned into the pGL4.21 luciferase expression vector (Promega, Madison, WI, USA). The vector was then subjected to in vitro methylation by using the M. SssI methyltransferase enzyme (Invitrogen, Grand Island, NY, USA). M. SssI recognizes the sequence pattern CpG and catalyzes the in vitro cytosine methylation at the recognized sequence pattern. Then, a luciferase assay was conducted in 293 T cell by using a Dual-Glo luciferase reporter assay system kit (Promega, Madison, WI, USA) 24 h after transfection. For the qPCR assays, the sequences of PCR primers are as follow: S100A12: forward primer (5′- CTTACAAAGGAGCTTGCAAAC-3′) and reverse primer (5′- GGTGTGGTAATGGGCAG-3′). 18S: forward primer (5′- GTAACCCGTTGAACCCCATT -3′) and reverse primer (5′- CCATCCAATCGGTAGTAGCG -3′).

### Cell culture, isolation and treatment

In this study, we cultured one leukocyte cell line (HL-60), one primary culture cell of human coronary artery endothelial cell (HCAEC) and one freshly isolated neutrophil. HL-60 (No. 60027, BCRC, Taiwan) was differentiated as neutrophil-like cell by the induction of 1.3% DMSO (Sigma-Aldrich, MO, USA). HL-60 and HCAEC (CC-2585, Lonza, Switzerland) were cultured and maintained as suggested by a previous study^22^. The freshly isolated neutrophils were collected from total blood of a healthy adult subject by using the Dynabeads CD15 (11137D, Invitrogen) as suggested by the manufacturer. In this study, neutrophils and HCAECs were treated with 20% serum, IVIG (3740501374, TBSF), recombinant S100A12 protein (1052-ER-050, R&D), antibody against S100A12 (PA5-76712, Invitrogen) and/or IgG control (UB276978, eBioscience). The administrated dosage of IVIG in culture medium is 10 mg/ml as suggested by a previous study^24^. The administrated dosages of S100A12, antibody and IgG control were all 152 ng/ml, approximately 20% of the mean of S100A12 serum concentrations in KD subjects (757.9 ng/ml, Table 3).

### Determining the intensities of surface adhesion molecules

The neutrophils treated with serum or S100A12 for 24 h were collected and washed with PBS. Then, they were stained with the following flow cytometry antibodies: CD11a-FITC (for ITGAL, 565280, BD), CD11b-FITC (for ITGAM, 563088, BD), CD18-FITC (for ITGB2, 743370, BD), CD29-FITC (for ITGB1, 746022, BD), CD49d-FITC (for ITGA4, 559,881, BD) or CD184-FITC (for CXCR4, 555974, BD). Next, they were quantified and analyzed with the LSRII flow cytometer (BD Biosciences). We used the geomeans to represent the intensities of the specific surface adhesion molecules.

### Leukocyte transendothelail migration (LTEM) assay

We used LTEM assay to evaluate the infiltration ability of neutrophils by referring to a previous study^22^. For this purpose, we prepared the LTEM assay by seeding 2 × 10^5^ HCAECs into gelatin-coated hanging inserts (also called the upper chamber, Merck, NJ, USA) for 24 h. In addition, we treated neutrophils (HL-60 cell line induced by DMSO) with serum, IVIG, recombinant S100A12 protein, antibody against S100A12 and/or IgG control for 24 h. For the fragile freshly isolated neutrophils, only four-hour treatment was applied.

On the day of the migration assay, the treated neutrophils were collected by serum-free medium wash. Then, 1 × 10^5^ neutrophils were placed in the inserts and the inserts were further moved into 24-well culture plates (also called the lower chamber) which contained 600 μl of medium with 200 nM fMLP (Sigma-Aldrich, MO, USA) as a chemo-attractant. After two-hour migration, the neutrophils penetrating the endothelial layer and migrating into the lower chamber were collected. The collected cells were then washed with PBS and stained with CD15-FITC (340703, BD), followed by quantification and analysis with the LSRII flow cytometer (BD Biosciences).

### Ethics statement

This study was approved by the institutional ethics board of KVGH with IRB approval number VGHKS19-CT2-22. All subjects or their guardians signed the informed consent form.

## Results

### Subject information

In this study, we enrolled healthy controls (HC), fever controls (FC, subjects with fever but not diagnosed as KD) and KD subjects. Table 1 showed that no significant age or gender difference was observed among the three sets of subjects. All KD subjects met the AHA 2004^25^ or JCS 2008 diagnostic criteria.

**Table 1:** Demographic table.

	HC (n = 37)	FC (n = 28)	KD (n = 78)	p-value		
				KD vs. HC	KD vs. FC	FC vs. HC
Age	1.98 ± 1.29	2.12 ± 1.23	1.58 ± 1.18	0.12	0.07	0.71
Gender (male)	48.65%	50%	51.28%	0.79	0.91	0.91

We enrolled healthy controls (HC), fever controls (FC) and KD subjects. The age and gender information of the three sets of subjects were tabulated. Among the three sets, the t-test (age) and chi-square test (gender) showed no significant difference on the age and gender factor, respectively.

### iTRAQ gel-free proteomics globally screened serum proteins

**Figure 1:**
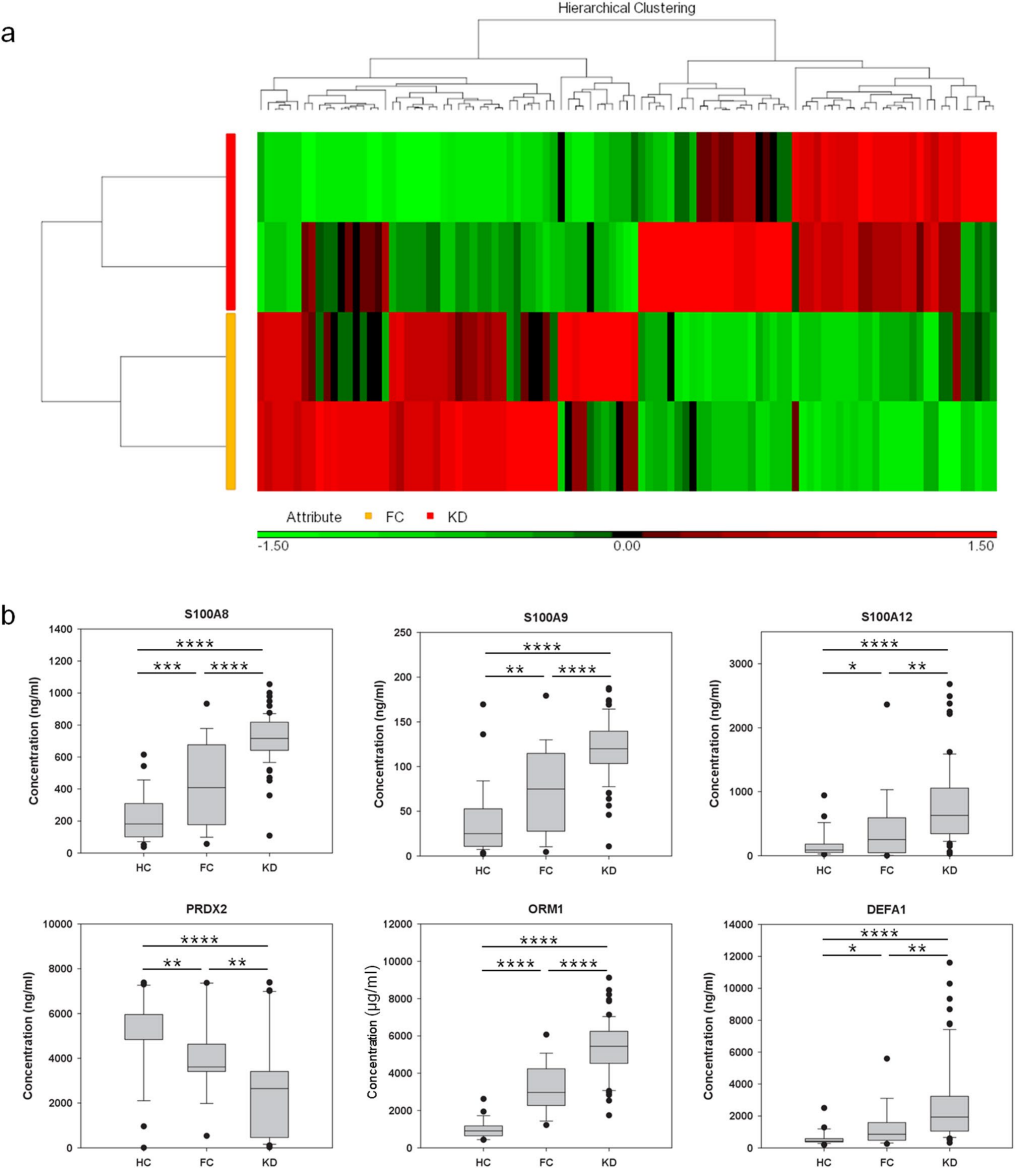
The serum protein profiles in samples. (**a**) We used iTRAQ gel-free proteomics to determine the relative intensities of serum proteins. Only 101 proteins with an average variation larger than 1.25-fold were presented in this heat map. (**b**) The FC and KD subjects were subjected to drawing blood after evident diagnosis and before treatment. Among these varied proteins, S100A8, A9 and A12 were selected for further ELISA validation owing to their inflammatory roles^56^. DEFA1, PRDX2 and ORM1 were also included, since they were linked to the GO functions (Table 2) and their abundance tendencies were reproducible for the two pooled FC and two pooled KD serum samples. *, **, *** and **** denoted p-values < 0.05, 0.01, 0.001 and 0.0001 according to t-tests, respectively.

We initially analyzed two pooled FC and two pooled KD serum samples with iTRAQ gel-free proteomics. As a result, we detected 372 proteins (Supplementary Table 1) in these samples according to two specific parameters (the parameters of Proteome Discoverer v2.4): the FDR for protein & peptide identification < 0.01 and the number of protein match unique peptide ≧1. We then inputted the protein abundance table into Partek software (Version: 7.0, https://www.partek.com/, Qiagen, Germany) and conducted ANOVA (KD vs. FC). As shown in Fig. 1a, we identified 101 proteins with variation greater than 1.25-fold. Figure 1a also showed that almost half of these proteins had higher abundance levels in FC samples, while the remaining half was more abundant in the KD samples.

To evaluate the reliability of the proteomic results and to derive the possible functions of these varied proteins, we also conducted GO analysis. Table 2 demonstrated that seven of the ten most significant GO items were inflammation, immune or stress-related functions, which was consistent with the fact that KD is an acute inflammatory disease. In addition, KD was also characterized with oxidative stress^26,27^. Therefore, iTRAQ gel-free proteomics resulted in a reliable dataset deserving further investigations.

**Table 2:** GO analysis.

Function	Type	p-value	# Genes	GO ID
Acute inflammatory response	BP	2.14E−16	12	2,526
Acute-phase response	BP	7.80E−15	10	6,953
Defense response	BP	9.51E−15	33	6,952
Response to stress	BP	1.53E−13	45	6,950
Response to wounding	BP	2.16E−13	21	9,611
Response to inorganic substance	BP	3.42E−13	18	10,035
Inflammatory response	BP	7.61E−13	18	6,954
Regulation of biological quality	BP	1.39E−11	40	65,008
Antioxidant activity	MF	9.54E−11	9	16,209
Response to stimulus	BP	1.21E−10	65	50,896

We conducted GO analysis on the 101 varied proteins shown in Fig. 1a. Only the ten most significant GO items were tabulated. BP and MF denoted the biological process and molecular function, respectively. The p-value was calculated based on the hypergeometric distribution. #gene denoted the number of the initially inputted gene that belonged to this function item. Since we were studying the serum proteins secreted out to the cells, the GO item cellular compartment did not receive further consideration.

### ELISA specifically validated KD biomarkers

Among the proteins detected by proteomics, we selected S100A8, S100A9, S100A12, PRDX2, ORM1 and DEFA1 for further ELISA validation in HC, FC and KD serum samples. The FC and KD subjects were subjected to drawing blood after evident diagnosis and before treatment (acute phase for KD). The statistical values of the protein concentrations were tabulated in Table 3. Figure 1b showed that the concentrations of all examined proteins significantly varied between the HC and FC samples and also between the FC and KD samples. In addition, five of the six examined proteins showed a significant concentration gradient, gradually increasing from the HC to FC samples and also from the FC to KD samples. However, the abundance tendency of PRDX2 was reversed to those of the other five proteins, gradually decreasing from the HC to FC samples and from the FC to KD samples. In summary, the six validated proteins may serve as KD biomarkers.

**Table 3:** The values of the concentrations in samples.

	S100A8		S100A9		S100A12		PRDX2		DEFA1		ORM1	
	Mean	SD	Mean	SD	Mean	SD	Mean	SD	Mean	SD	Mean	SD
HC	226.6	147.8	41.6	37.7	173.1	209.3	4,836.0	1,802.2	607.8	445.1	1,159.8	596.0
FC	434.5	231.3	75.6	46.3	405.5	544.3	3,610.6	1,653.8	1,344.6	1,510.7	3,222.2	1,229.3
KD	708.4	154.2	116.8	33.5	757.9	594.4	2,645.6	2,327.9	2,643.7	2,482.2	5,345.3	1,416.4
Pre-IVIG	717.8	137.7	117.3	31.3	769.2	588.9	2,809.8	2,287.7	2,596.6	2,316.6	5,328.3	1,455.4
Post-IVIG	206.7	62.9	22.7	15.4	387.3	384.6	2,323.3	1,431.2	1,639.7	1,362.5	4,714.4	1,542.3
Convalescent	76.1	51.0	9.5	12.5	142.2	178.2	2,670.7	1,276.6	350.6	320.9	1,068.0	563.5

We used ELISA to determine the concentrations of s in serum samples. There are 37, 28, 78, 63, 63 and 63 samples in the HC (healthy control), FC (fever control), KD, pre-IVIG (KD subjects at the acute phase without IVIG treatment), post-IVIG (KD subjects three days after IVIG treatment) and convalescent (KD subjects three weeks after IVIG treatment) sets, respectively. Since only 63 of the 78 KD subjects were followed after IVIG administration, the pre-IVIG set is a subset of KD set. For ORM1, the unit is µg/ml; for the others, the unit is ng/ml.

### Deriving a protein-based KD prediction model

**Figure 2:**
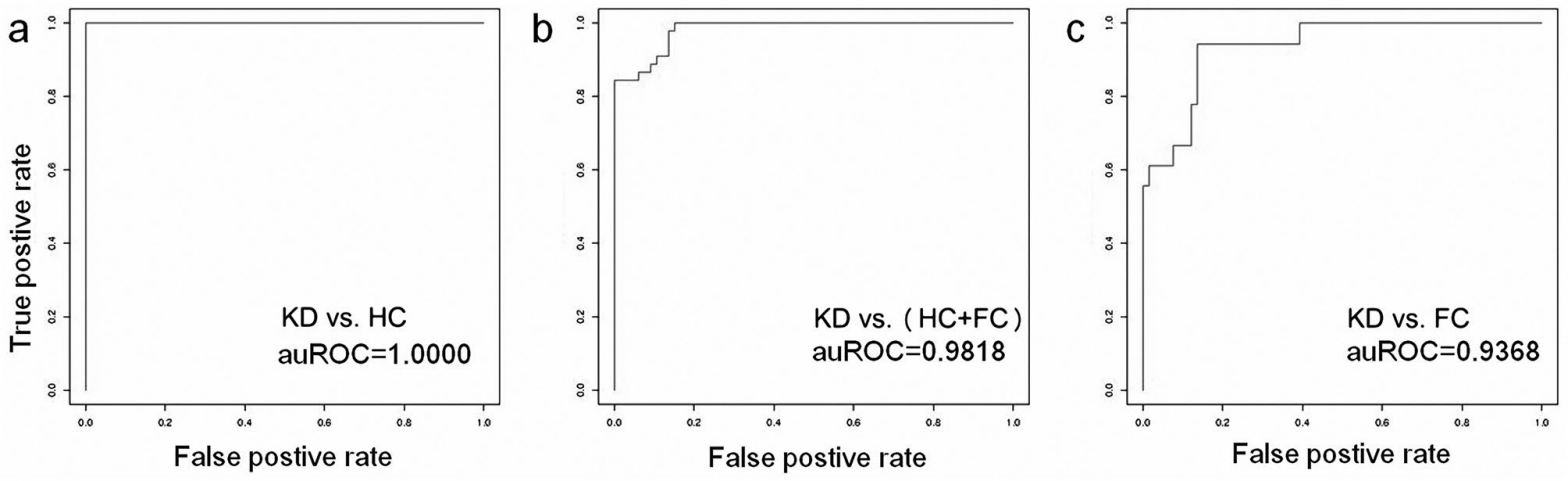
The performances of the derived KD prediction models. We used a type of machine learning algorithm, support vector machine (SVM), to derive the prediction models. The KD subjects were used to discriminate from (**a**) HC, (**b**) HC + FC and (**c**) FC subjects, resulting in different auROC values.

We further used the concentrations of the six protein biomarkers to derive KD prediction models with one type of machine learning algorithm, support vector machine (SVM, lib-svm version 3.22) to conduct the binary classification jobs as previous studies^21,28^ . In summary, we first conducted tenfold cross validation to derive the best parameters, gamma = 0.03125 and cost = 4. Then, by specifying these two parameters, we used all data points (the concentrations of six proteins in all samples) to train the prediction models. As shown in Fig. 2, the auROC values reached 1.0000, 0.9818 and 0.9368 when the KD set was compared with the HC, HC + FC and FC sets, respectively. Taking the KD vs. FC model for illustration, it had a sensitivity of 0.93 and a specificity of 0.86. When an independent cohort (15 KD and 10 FC samples, randomly isolated from the initial sample set) was used for validation, the prediction model reported a similar performance with a sensitivity of 0.93 and a specificity of 0.90. Such a model may facilitate the timely identification of KD and enable pediatric physicians and researchers to make preparations in advance.

### Concentrations of proteins varied with IVIG administration

**Figure 3:**
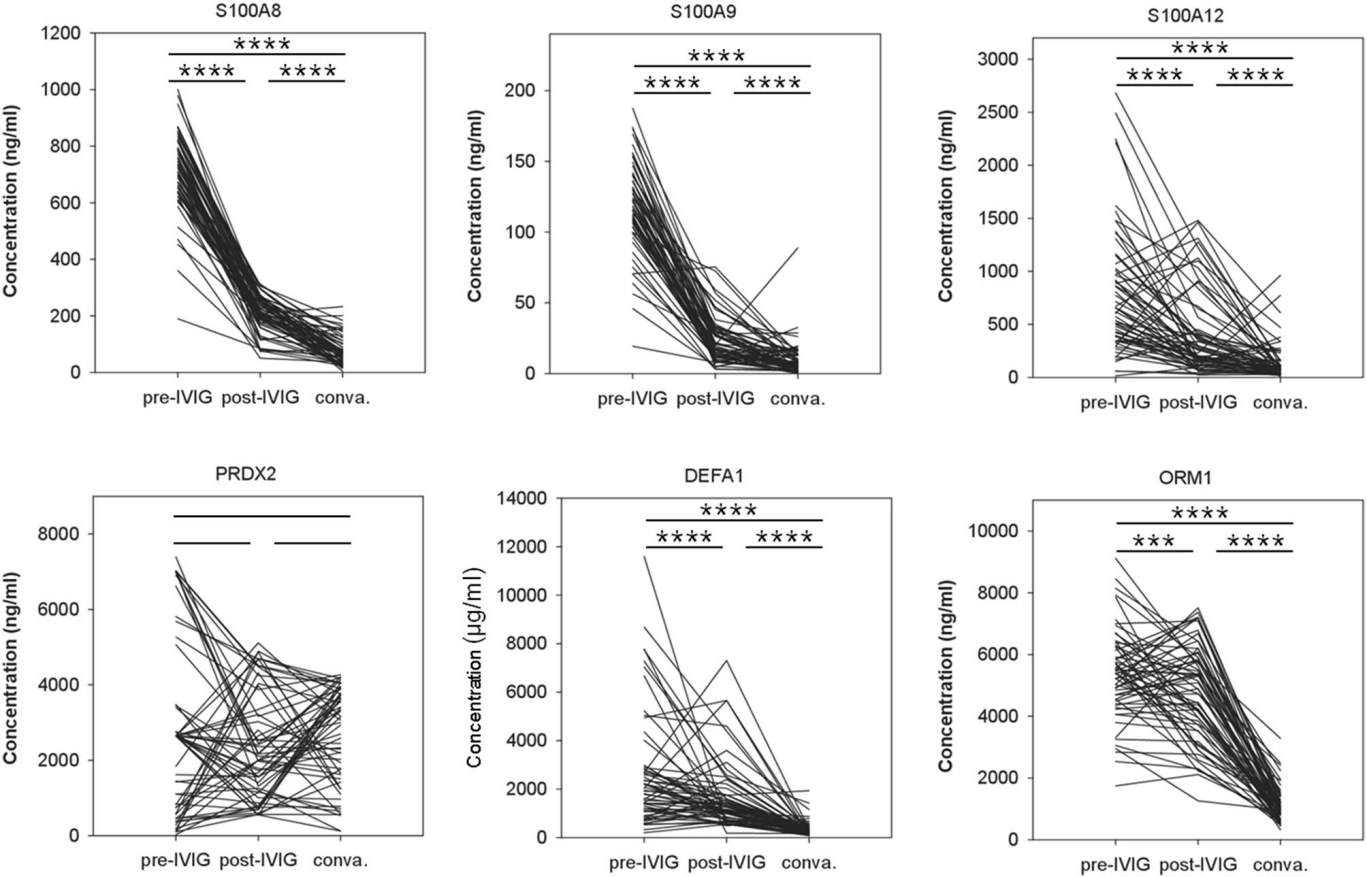
The variations of the six serum proteins before and after IVIG administration. Most of the KD subjects were subjected to ELISA for three times: at the acute phase before IVIG administration (pre-IVIG phase), three days after IVIG administration (post-IVIG phase) and three weeks after IVIG administration (convalescent phase). Each line presented the three ELISA values of one subject at the three phases. The sample size is 78. *** and **** denoted p-values < 0.001 and 0.0001 according to pair-wised t-tests, respectively.

Thus far, for the KD subjects, the concentrations of these KD biomarkers were determined only at the acute phase, namely before IVIG administration (pre-IVIG phase). We further examined their variations three days (post-IVIG phase) and three weeks (convalescent phase) after IVIG administration. As shown in Fig. 3, the five proteins with higher levels in the pre-IVIG set gradually and significantly decreased over time after IVIG administration, which highlights the efficacy of IVIG in treating systemic inflammation. Although the overall pattern reflected significant declines (e.g. for S100A12 and ORM1), the concentrations of several proteins increased after IVIG administration (post-IVIG) in many samples. We used Chi-square to compare whether decline or not was associated with CAL formation or IVIG non-responsiveness. It turned out that no significant difference was observed.

For PRDX2, its concentration before and after IVIG administration did not significantly vary. The statistical values of the protein concentrations in the three sets were also available in Table 3. According to Table 3, it seemed that the variations of S100A8, S100A9 and DEFA1 overreacted. At the convalescent phase (three weeks after IVIG administration), the concentrations of S100A8, S100A9 and DEAF1 declined to the levels even significantly lower than those of the HC set, lower levels than the healthy control subjects (Supplementary Fig. 1). Such decline owing to IVIG administration deserves further attention.

### S100A12 expression is activated by DNA hypomethylation

Among the six examined serum proteins, we were interested in S100A12.

**Figure 4:**
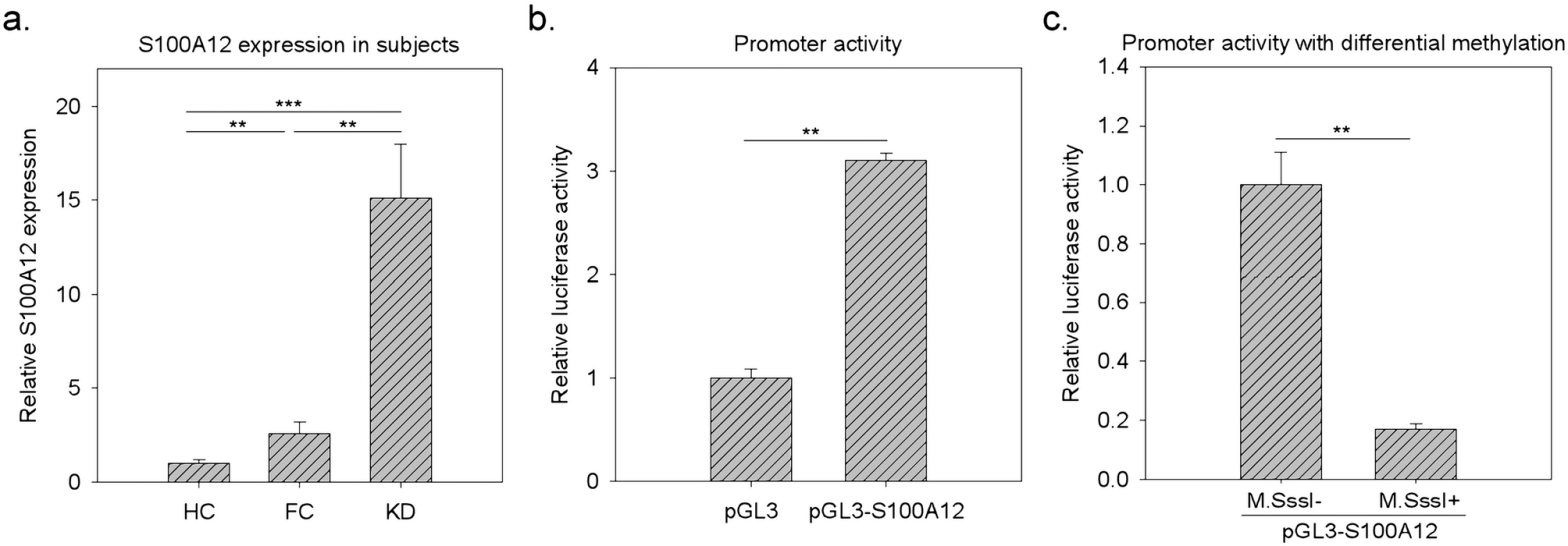
The variations of S100A12 gene expression and the promoter methylation assay. (**a**) We used qPCR to examine the relative mRNA levels of S100A12 in total WBCs as suggested by a previous study^22^. The variation tendency of the S100A12 gene in the WBCs was consistent with that of the S100A12 protein in serum. Data was presented as 2^-ΔΔCt^ with 18S gene as internal control. (**b**) Compared with empty pGL3 vector, pGL3-S100A12 demonstrated promoter activity. (**c**) The construct carrying the S100A12 promoter and luciferase was treated with M.SssI (totally methylated) or untreated (non-methylated), followed by transfection and luciferase assays. Data were presented as the mean ± SD. ** and *** denoted p-values < 0.01 and 0.001 according to t-tests, respectively.

S100A12 gene is usually highly expressed in neutrophils at acute phase of inflammation^29^. The serum soluble form of S100A12 protein is also elevated in many inflammation-related diseases^30,31^. Although S100A12 was also reported to be elevated in KD patients^32,33^, it’s pathogenesis role in KD was not clearly investigated. In our previous study^22^, the mRNA level of S100A12 kept higher in the WBCs of KD subjects than in those of HC and FC subjects. In addition, an in vivo evidence showed that the promoter region of S100A12 was hypomethylated in the KD samples. In this study, we also examined the mRNA level of S100A12. As shown in Fig. 4a, the mRNA level of S100A12 was significantly higher in KD samples than in FC and HD samples. Then, we further conducted a promoter methylation assay. As shown in Fig. 4b, the cloned upstream DNA fragment of S100A12 demonstrated promoter activity. Further, M.SssI treatment (M.SssI+), compared with non M.SssI treatment (M.SssI-), caused total DNA methylation and subsequently decreased luciferase activity (Fig. 4c). Huang et al. and this study provided in vivo and in vitro evidences, respectively, that S100A12 expression was really regulated by DNA methylation.

### S100A12 activated the adhesion molecules of neutrophils

**Figure 5:**
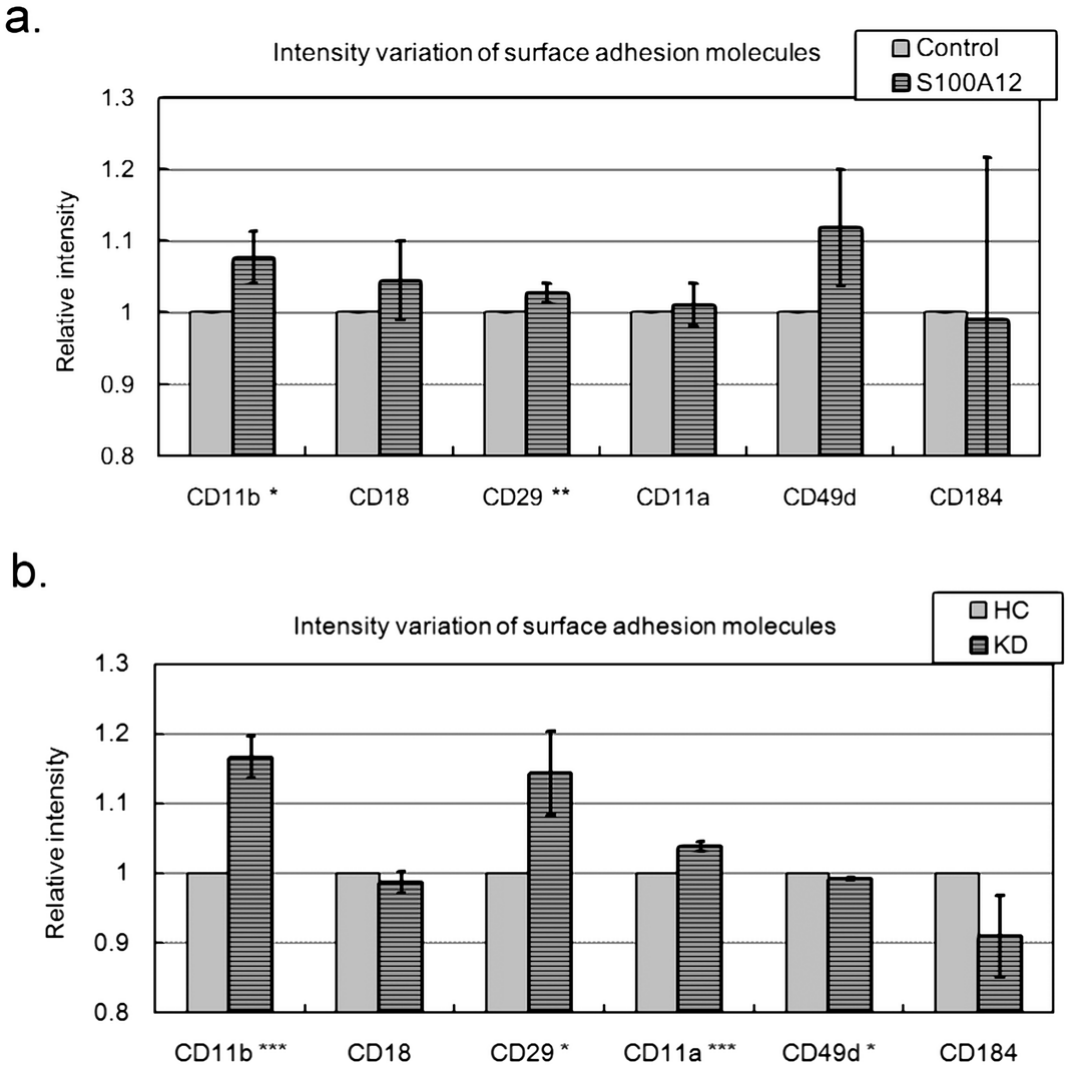
The variations of the surface adhesion molecules anchored on the surfaces of leukocytes. We used flow cytometry to determine the intensities (geo-means) of the surface adhesion molecules of leukocytes. (**a**) Neutrophils were treated with S100A12 or not for 24 h and then measured with flow cytometry. (**b**) Neutrophils were treated with KD serum (evenly pooled serum samples) or HC serum (evenly pooled serum samples) for 24 h and then measured with flow cytometry. Data were presented as mean ± SD. *, ** and *** denoted p-values < 0.05, 0.01 and 0.001 according to t-tests, respectively.

In our previous study^22^, we concluded that neutrophils treated with S100A12 recombinant protein promoted neutrophil infiltration through the HCAEC layer. Since adhesion between leukocytes and endothelail cells is a critical step of leukocyte infiltration^34^, we examined whether S100A12 activated the six adhesion molecules anchored at the surfaces of leukocytes^35^. As shown in Fig. 5a, with S100A12 treatment, the intensities (according to geo-means determined with flow cytometry) of CD11b (ITGAM) and CD29 (ITGB1) were significantly enhanced. Although significant, the enhanced variations of CD11b and CD29 were pretty slight, as they were both less than 10%. Therefore, we repeated the assays by replacing S100A12 treatment with serum treatment (20% pooled KD or HC serum samples). Figure 5b showed that KD serum, compared with HC serum, brought result similar to those of S100A12 treatment. In addition, KD serum treatment enhanced the intensities of CD11b and CD29 by 17% at most. Therefore, the observed values in Fig. 5a were reasonable.

### Antibody attenuated the in vitro neutrophil infiltration promoted by S100A12

**Figure 6:**
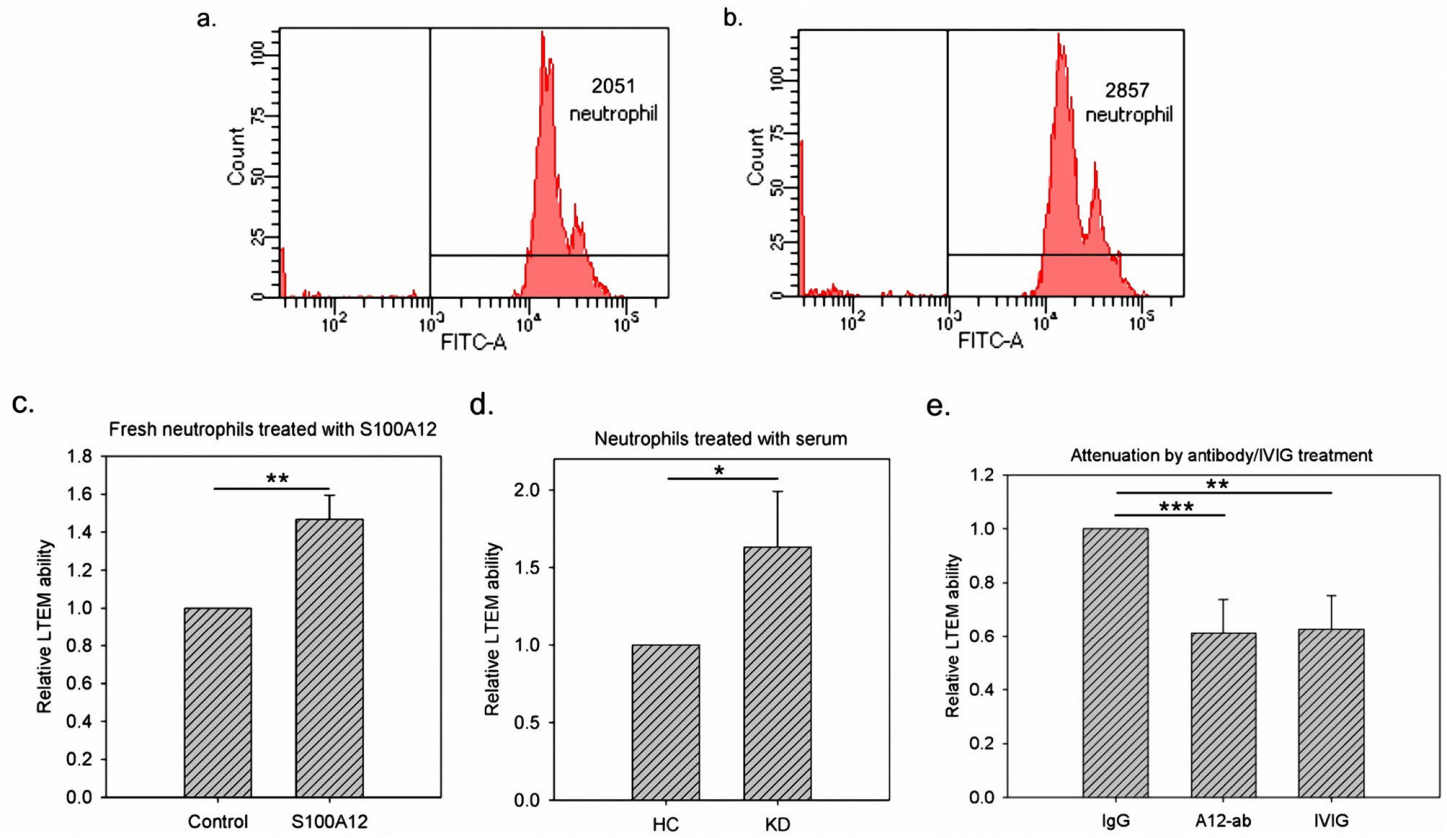
The results of LTEM assays with neutrophils manipulated. (**a**,**b**) Clinically freshly isolated neutrophils were left untreated or treated with S100A12, followed by LTEM assays. Compared with the control, S100A12 treatment allowed more neutrophils to penetrate the endothelial layer (2,857 vs. 2,051). (**c**) By three independent assays, S100A12 treatment significantly promoted freshly isolated neutrophils (donated by a healthy male adult) to infiltrate and to migrate through the endothelial layer. (**d**) KD serum promoted neutrophils (cell line) infiltration by approximately 1.7-fold, which allowed us to mimic KD disease by using serum treatment (n = 3). (**e**) KD serum-treated neutrophils (cell line) were simultaneously treated with a control IgG, S100A12 antibody or IVIG, followed by LTEM assays (n = 3). Data were presented as the mean ± SD. *, **, *** and **** denoted p-values < 0.05, 0.01, 0.001 and 0.0001 according to t-tests, respectively.

The conclusions in our previous study^22^ were made by manipulating a neutrophil-like cell line (HL-60). We further repeated the leukocyte transendothelial migration (LTEM) assay with freshly isolated neutrophils donated by a healthy adult male volunteer. As shown in Fig. 6a,b, 2,051 and 2,857 freshly isolated neutrophils penetrated the endothelial layer without and with S100A12 treatment, respectively. By three independent assays, S100A12 treatment significantly promoted freshly isolated neutrophils to migrate through the endothelial layer by 1.5-fold (Fig. 6c, fresh neutrophils donated by a healthy male adult).

Next, we examined whether S100A12 antibody may attenuate the infiltration promoted by S100A12. We first mimicked KD by using KD serum treatment. Similar to S100A12 treatment, compared with HC serum, KD serum significantly promoted neutrophil infiltration by approximately 1.7-fold (Fig. 6d). This promotion was attenuated when antibody was added to neutralize the endogenous S100A12 protein in KD serum (Fig. 6e). In addition, IVIG treatment had the similar effect as S100A12 antibody did (no difference observed between S100A12 and IVIG). In summary, the antibody against S100A12 may attenuate the in vitro neutrophil infiltration similar to IVIG.

## Discussion

In this study, we evaluated neutrophil infiltration with an in vitro LTEM model. Most assays have focused on S100A12’s effects on neutrophils and concluded that recombinant S100A12 protein and antibody promoted and attenuated neutrophil infiltration, respectively. Such promotion could result from activating neutrophil surface adhesion molecules, enhancing the adhesion between neutrophils and endothelial cells. Previous studies have attempted to clearly define the clinical and epidemiologic characteristics of KD, facilitating precise disease identification^36,37^. In addition, other studies have also identified molecular biomarkers of KD, including those involved in gene expression profiles^28,38^ and DNA methylation^39^ . However, previous RNA-related studies utilized fragile RNA samples from leukocytes, which required troublesome and lengthy processing. The DNA methylation-related study required long periods of waiting for bisulfite conversion and DNA re-sequencing. Therefore, the time-saving ELISA methodology is more suitable for identifying KD. In addition, compared with DNA and RNA from leukocytes, serum samples are easy to store and ship, enhancing the applicability of the entire procedure.

Although serum protein-based biomarkers of KD onset and/or complications were proposed previously, these studies needed advancement. They did not develop a diagnosis or prognosis model using the identified biomarkers, failing to predict KD onset or complications^40–42^. In some cases, although a prediction model was developed, the model performance was still behind satisfaction^43–45^. Moreover, these studies initiated by monitoring some specific cytokines or traditional proteomics^40–45^, which confined the potential biomarkers to a limited number of candidates. Therefore, novel biomarkers could not be identified, which is why we initiated the study by using iTRAQ gel-free proteomics to globally screen all serum proteins.

This study was initiated by enrolling subjects and analyzing blood samples. Therefore, the overall analysis results could be influenced by the racial factor. Actually, all subjects participating this study are Han Chinese, reflecting the major limitation of this study. With the subjects of more racial diversity undergoing ELISA on the six KD biomarkers, we may derive a more unbiased and robust KD prediction model. Another weak point of this study is without the support from in vivo animal model. S100A12 gene has no orthologous gene in mouse. Manipulating S100A12 protein or gene in mouse is not reasonable, so that this weak point seems to be unavoidable.

Since S100A12 promoted in vitro neutrophil infiltration which is the cause of in vivo CAL formation, we also examined whether the abundance of serum S100A12 varied with CAL formation. Among all KD patients participating this study, 16 subjects had CALs. We further compared S100A12 serum level between 16 KD subjects with CALs and the remaining KD subjects without CALs. It turned out that no significant difference was observed between the two sets (p = 0.109), although the in vitro cell assays implied S100A12’s role in CAL formation. Such inconsistency deserves further investigations in the future.

Lipopolysaccharide (LPS) is usually applied in animal model to induce systemic inflammation^46,47^ and also applied in leukocytes or other cells to induce inflammatory reactions or cell damage^48,49^. However, LPS is seldom applied in neutrophils to examine transendothelial migration. We also treated neutrophils with LPS by 152 ng/ml (low dosage, as S100A12 recombinant protein) and 1 μg/ml (high dosage, as suggested by other studies), followed by the same assays shown in Fig. 6c. Compared with control set (no LPS treated), no significant variation of relative LTEM ability was observed no matter low or high dosage LPS was treated. Therefore, LPS is not suitable for developing an in vitro leukocyte infiltration model.

In this study, we observed that S100A12 antibody and IVIG had similar effects on attenuating in vitro neuthophil infiltration. S100A12 antibody functioned by specifically inhibiting serum S100A12 protein molecules. IVIG is the standard treatment of KD and its best administration is within 10 days of KD onset at a single dose of 2 g/kg infusion over 10–12 h^2^. The mechanisms of IVIG in reducing in vivo and in vitro neuthophil infiltration are still unclear. Possible mechanisms include Fc receptor blockade, neutralization of the infectious agents or toxins, modulating functions of T and B cells, induction of suppressor activity, and modulation of the production of cytokines and cytokine antagonists^50^. In this study, the used dosages of S100A12 antibody and IVIG (Method section) were different so that it is difficult to tell which molecule worked better.

IVIG is also thought to be a cause of cytokine storm, greatly influencing variation and balance in individuals. Although IVIG is a popular and powerful therapy for many autoimmune diseases and KD, it has been reported to have many side effects^51–55^. In this study, we used ELISA to monitor the variations of KD protein biomarkers. As shown in Fig. 3, the levels of most KD biomarkers recovered after IVIG administration. However, the levels of S100A8, S100A9 and DEFA1 in the convalescent phase (three weeks after IVIG administration) declined so that they were significantly lower than in HC subjects (Supplementary Fig. 1). The question of whether such a decline to levels lower than those in HC subjects is harmful or not deserves further investigation.

## Conclusion

In this study, we first conducted global screening and specific validation on serum proteins in Kawasaki disease (KD). As a result, we identified six KD protein biomarkers which enabled us to develop a high-performance KD prediction model. Next, we found that the levels of the six KD biomarkers gradually recovered after IVIG administration. Among the six KD biomarkers, we further investigated S100A12 and concluded that S100A12 gene expression was truly regulated by DNA methylation. Finally, we found that S100A12 promoted neutrophil infiltration through the endothelial layer by affecting neutrophils. And, such promotion can be attenuated by the antibody against S100A12. Therefore, the antibody against S100A12 has the potential to serve as an alternative therapy to augment traditional IVIG administration.

## Supplementary information

Supplementary file1
