# Supplementary material for: Serum proteins may facilitate the identification of Kawasaki disease and promote in vitro neutrophil infiltration
Source: Sci Rep. 2020 Sep 24;10:15645. doi: 10.1038/s41598-020-72695-z (PMC7518260; doi:10.1038/s41598-020-72695-z)
Supplement: Supplementary file 1 — Supplementary file1 [file 41598_2020_72695_MOESM1_ESM.pdf]

Serum proteins may facilitate the identification of Kawasaki disease and promote *in vitro* neutrophil infiltration

Sung-Chou Li[1,*], Kuo-Wang Tsai[2,*], Lien-Hung Huang[3,*], Ken-Pen Weng[4,5,6,#], Kuang-Jen Chien[4], Yuyu Lin[1], Chi-Ying Tu[4], Pei-Hsien Lin[1]

1 Genomics and Proteomics Core Laboratory, Department of Medical Research, Kaohsiung Chang Gung Memorial Hospital and Chang Gung University College of Medicine, Kaohsiung, Taiwan
2 Department of Research, Taipei Tzu Chi Hospital, Buddhist Tzu Chi Medical Foundation, New Taipei, Taiwan
3 Department of Neurosurgery, Kaohsiung Chang Gung Memorial Hospital and Chang Gung University College of Medicine, Kaohsiung, Taiwan
4 Congenital Structural Heart Disease Center, Department of Pediatrics, Kaohsiung Veterans General Hospital, Kaohsiung, Taiwan
5 Department of Medicine, National Yang-Ming University, Taipei, Taiwan
6 Shu-Zen Junior College of Medicine and Management, Kaohsiung, Taiwan

* Equal contribution
# Address requests for reprints/correspondence to:
Ken-Pen Weng: Congenital Structural Heart Disease Center, Department of Pediatrics, Kaohsiung Veterans General Hospital, Kaohsiung, No.386, Dazhong 1st Rd., Zuoying Dist., Kaohsiung, Taiwan, Tel: 011-886-7-346-8203, Fax: 011-886-7-346-8207

E-mail addresses: SCL: raymond.pinus@gmail.com; KWT: kwtsai6733@gmail.com; LHH: ahonbob@gmail.com; KPW: kenpenweng@yahoo.com.tw; KJC: kjchien@vghks.gov.tw; YL: linyuyu030@gmail.com; CYT: chiyingofficial@gmail.com; PHL:

jio4872@gmail.com;

Running title: S100A12's pathogenic roles in KD

Supplementary Figure 1. Cytokine variations among different subjects and with IVIG administration. To compare the cytokine variations, we integrated the data of Figure 1b and Figure 3 into Table 3. To provide a comprehensive comparison, we plotted the variations of three serum proteins. After three weeks of IVIG administration (convalescent set), the concentrations of S100A8, S100A9 and DEFA1 significantly declined even lower than those of the HC set (p-value= 1.223E-10, 2.418E-08 and 0.001 for S100A8, S100A9 and DEFA1, respectively). The sample sizes of the HC, FC KD, pre-IVIG, post-IVIG and convalescent sets are 37, 28, 78, 63, 63 and 63, respectively. Data were presented as the mean±SEM.

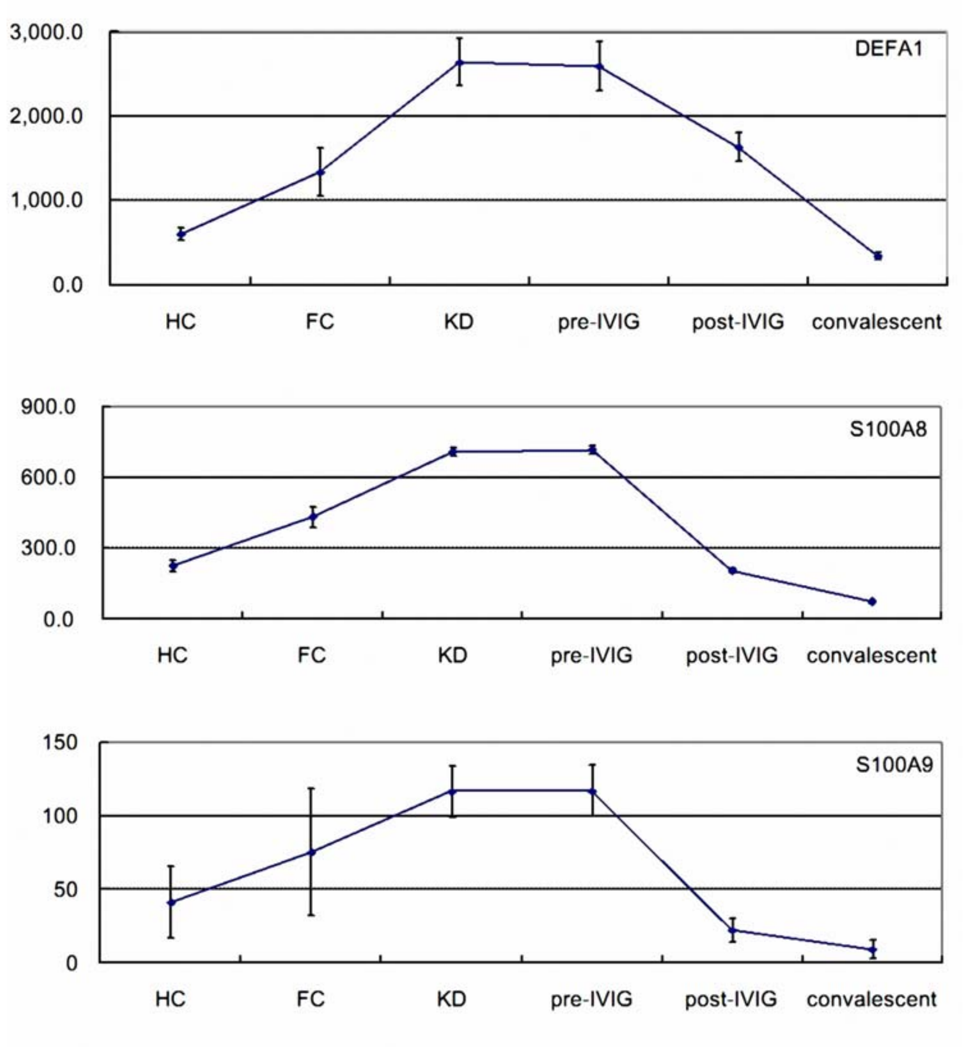

3,000.0
2,000.0
1,000.0
0.0
DEFA1
HC
FC
KD
pre-IVIG
post-IVIG
convalescent
900.0
600.0
300.0
0.0
S100A8
HC
FC
KD
pre-IVIG
post-IVIG
convalescent
150
100
50
0
S100A9
HC
FC
KD
pre-IVIG
post-IVIG
convalescent

Supplementary Table 1. The abundances of serum proteins quantified by iTRAQ. We had two pooled FC and two pooled KD serum samples analyzed with iTRAQ. The abundances of these serum proteins were shown in this table.

Accession	Exp. q-value	Coverage	# Protein Groups	# Peptides	# Unique Peptides	# PSMs	MW [kDa]	Abundances: 114-KD	Abundances: 115-FC	Abundances: 116-KD	Abundances: 117-FC
P98160	0.0000E+00	0.9793	1	2	2	2	468.5	6.6	11.1	11.8	13.7
P59665	5.0000E-03	32.9787	1	1	1	1	10.2	9.2	5.8	7.2	2.8
Q6UWP8	5.0000E-03	9.1525	1	1	1	2	60.5	9.6	11.9	10.6	12.8
Q01518	6.0000E-03	3.3684	1	1	1	1	51.9	12.9	9.2	7.2	8.6
P00488	6.0000E-03	3.4153	1	1	1	1	83.2	13.7	10.1	12.6	14.2
P13716	3.0000E-03	5.7576	1	1	1	1	36.3	13.8	12.1	10.9	12.6
P06326	6.0000E-03	9.6000	1	1	1	1	13.6	14.3	12	10.7	12.1
P60900	6.0000E-03	8.5366	1	2	2	2	27.4	14.6	14.8	18.3	22.3
Q15166	0.0000E+00	7.3446	1	3	1	5	39.6	15.7	32.3	23	22
Q9H4G4	6.0000E-03	11.0390	1	1	1	1	17.2	16.2	13.4	25.4	18.6
P02788	0.0000E+00	5.2113	1	3	3	3	78.1	16.3	20	20.5	19.5
Q04721	6.0000E-03	0.8094	1	1	1	1	265.2	16.7	14.6	16.5	13.4
O75144	3.0000E-03	5.9603	1	1	1	1	33.3	17.1	18.8	14	15.4
Q9Y490	3.0000E-03	0.9839	1	2	2	2	269.6	17.9	19.2	25.4	28.2
Q99497	6.0000E-03	4.7619	1	1	1	1	19.9	18.1	26	36.9	43.3
Q6UY14	3.0000E-03	3.2588	1	2	2	2	116.5	18.3	14.8	17.4	14.4

P69892	0.0000E+00	89.7959	1	12	1	28	16.1	18.7	88.3	23.2	25.1
P01625	3.0000E-03	28.9474	1	2	2	2	12.6	20.2	13.6	15	14
Q16270	3.0000E-03	10.6383	1	2	2	2	29.1	20.7	27.2	26.9	22.7
Q13228	0.0000E+00	10.5932	1	3	3	3	52.4	20.8	26.9	27.6	34
P01593	3.0000E-03	16.6667	1	1	1	2	12	20.8	16.2	15.2	12.8
Q9H4A9	3.0000E-03	5.7613	1	2	2	2	53.3	21	23.4	15.2	20.8
Q9HCN6	3.0000E-03	7.9646	1	2	2	2	36.8	21.8	28.2	29.5	30.8
Q13449	3.0000E-03	4.7337	1	1	1	1	37.4	22.1	21.2	19.6	14.5
P18669	6.0000E-03	6.2992	1	1	1	1	28.8	22.7	19.2	19.4	17.7
P62258	0.0000E+00	10.5882	1	2	1	2	29.2	22.8	29.8	32	39
P12821	6.0000E-03	0.8423	1	1	1	1	149.6	22.9	36.7	29.3	33.7
Q8NBP7	3.0000E-03	3.9017	1	2	2	2	74.2	23.3	25.3	31	27.6
Q9Y6R7	6.0000E-03	0.7771	1	2	2	2	571.6	24.7	23.1	29.8	25.8
Q12884	0.0000E+00	5.1316	1	3	2	5	87.7	25.8	29.3	24.9	27.1
O95497	0.0000E+00	12.2807	1	4	4	4	57	27.1	27.1	26.1	32.6
Q9HDC9	3.0000E-03	5.5288	1	2	2	2	46.5	27.3	21.9	22.6	21.6
P05019	0.0000E+00	14.8718	1	2	2	4	21.8	27.6	46.1	38.3	47.4
P05164	0.0000E+00	5.6376	1	3	3	3	83.8	27.6	25.3	24.3	18.9
O00299	6.0000E-03	4.9793	1	1	1	1	26.9	27.6	28	28.1	32.5
P22897	3.0000E-03	2.8846	1	4	4	4	165.9	28.1	29.7	32.3	26.8
P03950	5.0000E-03	10.8844	1	2	2	2	16.5	28.2	38.2	34.3	43.4
P08779	0.0000E+00	10.1480	1	6	2	6	51.2	28.6	18	26.1	11.1

Q9NTU7	6.0000E-03	5.9701	1	1	1	1	21.8	30.1	35.3	31	43.6
P12109	0.0000E+00	3.7938	1	3	3	3	108.5	30.3	42.3	32	42.4
P11142	0.0000E+00	9.7523	1	5	3	6	70.9	31.1	31.9	35.9	43.5
P01034	6.0000E-03	7.5342	1	1	1	1	15.8	31.8	24.9	24.1	35.3
P02452	0.0000E+00	2.6639	1	3	3	3	138.9	32	41.4	28	58.6
P35916	0.0000E+00	4.3287	1	3	3	3	152.7	32	30.3	32.4	28.8
P10451	6.0000E-03	5.4140	1	1	1	1	35.4	32.6	33.2	36.8	30.7
P10599	3.0000E-03	31.4286	1	3	3	3	11.7	32.7	27.6	33.4	40.2
P07195	0.0000E+00	10.7784	1	3	2	4	36.6	33.2	43.4	34.5	40.6
P68032	0.0000E+00	25.1989	1	8	1	25	42	33.5	27.7	42.7	33
P67936	0.0000E+00	13.7097	1	4	3	5	28.5	33.5	32.1	51.9	50.4
O14786	3.0000E-03	2.2752	1	2	2	2	103.1	34.4	39.1	47.5	46.9
Q86U17	6.0000E-03	5.6872	1	2	2	2	47	34.8	31.9	34.1	39
Q13201	0.0000E+00	4.8046	1	4	4	5	138	35.1	29.6	18.8	18
P07942	0.0000E+00	3.4155	1	4	4	4	197.9	35.5	32.8	41.2	36.6
P32942	6.0000E-03	3.2907	1	2	2	2	59.5	35.7	44.9	44.9	48.3
P23528	0.0000E+00	25.9036	1	3	3	3	18.5	36.3	35.5	43.9	45.5
P08294	0.0000E+00	22.5000	1	3	3	4	25.8	36.8	35.3	50	34.6
Q14766	6.0000E-03	1.1040	1	2	2	2	186.7	36.9	45.1	60.6	50.4
O00592	6.0000E-03	3.7634	1	2	2	2	58.6	37.5	49.6	34.4	40.6
P09172	0.0000E+00	9.8865	1	4	4	6	69	38.2	50.2	42.9	41.5
P54289	3.0000E-03	2.3572	1	3	3	3	124.5	38.3	47.6	43.5	47.3

P63313	5.0000E-03	47.7273	1	2	1	2	5	38.5	41.7	40.9	29.5
P01743	6.0000E-03	9.4017	1	1	1	1	12.9	39.6	28.4	22.1	25.5
P62158	0.0000E+00	23.4899	1	2	2	2	16.8	39.9	39.4	63.9	53
Q9H299	3.0000E-03	27.9570	1	2	2	2	10.4	40	53.9	59.3	48.5
P99999	5.0000E-03	18.0952	1	2	2	2	11.7	40.4	48.6	40.7	49.9
P81605	6.0000E-03	22.7273	1	2	2	2	11.3	40.7	85.4	45.7	50.1
P07237	0.0000E+00	8.4646	1	3	3	3	57.1	40.9	36.9	40.2	41.3
P15531	6.0000E-03	7.8947	1	1	1	1	17.1	41.1	63.3	62.2	71.3
P29401	0.0000E+00	7.3836	1	4	4	4	67.8	41.4	44.1	51.8	36.4
P78417	6.0000E-03	9.1286	1	2	2	2	27.5	41.6	40.9	43.6	49.3
P69891	0.0000E+00	89.7959	1	12	1	26	16.1	42	96.3	59.1	76.2
P10124	0.0000E+00	20.8861	1	2	2	3	17.6	42.5	32.8	47.1	39.8
Q99715	6.0000E-03	0.9794	1	2	2	2	332.9	42.8	48.9	46	43.4
P20742	0.0000E+00	11.7409	1	16	6	119	163.8	42.9	63.9	103.2	48.5
P17813	0.0000E+00	4.2553	1	2	2	2	70.5	42.9	50.8	49.8	79.4
P33151	6.0000E-03	2.0408	1	1	1	1	87.5	43.1	62	59.5	61.3
P07602	3.0000E-03	2.8626	1	1	1	1	58.1	43.2	42.9	40.6	39.1
P00746	0.0000E+00	33.2016	1	5	5	6	27	43.4	55.3	53.1	64
Q8N6C8	0.0000E+00	17.7677	1	6	6	8	47.4	43.6	94.3	97	54.4
Q14956	6.0000E-03	1.5734	1	1	1	2	63.9	44.3	68.8	57.8	71.8
O15394	0.0000E+00	6.2127	1	4	4	4	93	44.9	41.4	47.4	47.6
P62937	0.0000E+00	15.1515	1	3	3	3	18	45.3	56	64.7	75.1

P63104	0.0000E+00	41.6327	1	7	6	9	27.7	46.7	36	51.5	51.6
P07339	0.0000E+00	14.3204	1	4	4	5	44.5	47	41.8	44.6	43.1
Q15848	0.0000E+00	29.5082	1	4	4	10	26.4	47.6	59.8	61.4	70.6
P14625	0.0000E+00	3.1133	1	2	2	2	92.4	47.8	42.1	49.9	49.8
P20023	3.0000E-03	2.8074	1	3	3	3	112.8	48.2	64.7	43.8	55.6
P13942	6.0000E-03	0.9217	1	1	1	1	171.7	48.7	31.3	21.6	31.7
P23142	0.0000E+00	6.4011	1	3	3	3	77.2	49.2	52	57.8	51.1
P05062	0.0000E+00	12.9121	1	4	3	4	39.4	49.6	36.7	61.3	42
Q13740	0.0000E+00	8.0617	1	4	4	4	65.1	49.9	58.4	58	60.6
P30043	0.0000E+00	23.7864	1	3	3	4	22.1	50.1	68.7	65.8	84.7
P08519	0.0000E+00	16.3369	1	6	6	7	501	50.3	56.3	174.6	60.7
P21333	0.0000E+00	2.4556	1	6	6	6	280.6	50.3	51.7	74.9	72.2
P22891	0.0000E+00	9.7500	1	4	4	5	44.7	50.3	49.6	49.2	52.8
P06753	3.0000E-03	9.4737	1	3	2	3	32.9	50.7	44.3	49.8	44.7
Q12841	0.0000E+00	11.6883	1	3	3	3	35	51.8	54.9	50.8	52.6
Q6YHK3	0.0000E+00	2.8374	1	4	4	5	161.6	52	64.5	64.5	69.9
P07737	0.0000E+00	27.1429	1	3	3	3	15	52	48.2	54.3	50.2
P11717	0.0000E+00	2.3685	1	5	5	5	274.2	52.8	53.1	63.8	60.9
P01859	0.0000E+00	25.4601	1	6	3	10	35.9	53.1	44.9	49.8	40.7
P16112	0.0000E+00	1.2008	1	2	2	2	250	53.7	76.5	66.8	76.4
P62805	6.0000E-03	9.7087	1	1	1	1	11.4	54	59.2	105.9	56.5
P23470	3.0000E-03	1.5225	1	2	2	2	161.9	54.4	57.8	50.5	56.3

P04179	6.0000E-03	3.6036	1	1	1	1	24.7	55.7	34.4	57.8	42
Q9Y4L1	0.0000E+00	4.4044	1	3	3	4	111.3	56	56.9	55.9	49.5
P36980	0.0000E+00	18.5185	1	5	4	11	30.6	56.5	85.1	96.6	80.2
P60174	6.0000E-03	8.7413	1	2	2	2	30.8	56.9	91.6	70.5	88.1
P01860	0.0000E+00	29.1777	1	7	2	16	41.3	57.4	43.8	53.2	37.9
P27487	0.0000E+00	10.4439	1	7	7	8	88.2	57.5	66	60.7	63.9
Q12805	0.0000E+00	11.5619	1	5	5	5	54.6	58.8	63.7	78.2	75.1
Q9NPY3	0.0000E+00	4.4479	1	2	2	2	68.5	59	69.2	52.3	70.8
P0DJI9	0.0000E+00	63.9344	1	9	3	50	13.5	59.3	94.6	45.1	41.2
P12111	0.0000E+00	4.2493	1	11	11	11	343.5	60	69.4	65.7	75.8
Q76LX8	0.0000E+00	2.2425	1	3	3	3	153.5	60.2	83.1	87.6	112.1
P01033	0.0000E+00	14.9758	1	2	2	3	23.2	60.5	42	53.5	42.9
Q99784	0.0000E+00	8.6598	1	4	4	8	55.3	60.8	64.9	70.5	85.3
Q9UNW1	0.0000E+00	11.0883	1	4	4	4	55	62.1	71.6	62	68.6
P43121	5.0000E-03	4.9536	1	2	2	2	71.6	62.8	71.2	64.8	68.8
Q12913	0.0000E+00	6.0583	1	6	6	8	145.9	63	74.8	68.5	79.8
Q13822	0.0000E+00	7.6477	1	6	6	8	98.9	63.5	58.3	71.1	72.7
P24043	8.0000E-03	0.5766	1	2	2	2	343.7	64.4	59.7	62.4	64.5
P20848	6.0000E-03	3.3333	1	2	1	25	47.9	64.5	23.3	111.8	28.4
P35555	0.0000E+00	1.8112	1	4	4	4	312	64.8	66.3	64.6	77.3
P00390	5.0000E-03	3.6398	1	2	2	2	56.2	65.3	90.2	79.6	82.8
Q92496	0.0000E+00	5.7093	1	3	3	3	65.3	65.4	62.7	74.3	72.8

Q86Y46	6.0000E-03	3.8889	1	2	1	2	58.9	65.4	59	77.6	56.9
P49747	0.0000E+00	29.0621	1	14	11	21	82.8	66.5	72.6	76.7	99.9
P00338	0.0000E+00	18.9759	1	6	5	6	36.7	66.8	89.6	87.7	95.9
Q6UX71	3.0000E-03	5.2930	1	3	3	3	59.5	66.8	66.4	65.6	66.1
P61769	0.0000E+00	35.2941	1	3	3	6	13.7	66.9	69	55.3	81.2
Q14314	3.0000E-03	3.4169	1	2	2	2	50.2	66.9	95.3	85.2	81.3
Q9UHG3	3.0000E-03	4.7525	1	3	3	3	56.6	67.1	65.2	97.4	64.4
P13598	0.0000E+00	11.6364	1	3	3	5	30.6	67.4	95.5	89.9	97.8
P04220	0.0000E+00	46.0358	1	12	1	39	43	68.5	31	40.2	29.4
Q9BY67	0.0000E+00	10.1810	1	3	3	3	48.5	70.4	65.8	81.3	65
P01765	0.0000E+00	26.0870	1	2	2	2	12.3	70.9	52.8	54.4	54.8
Q14515	0.0000E+00	5.2711	1	3	3	4	75.2	71.4	95.7	83.6	98.4
O00533	0.0000E+00	9.9338	1	9	9	11	135	71.5	88	76.9	81
Q13103	0.0000E+00	26.5403	1	5	5	12	24.3	72.4	70.4	73	95.6
P00748	0.0000E+00	11.8699	1	5	5	7	67.7	72.4	73.3	86	79.3
P62979	0.0000E+00	16.0256	1	2	2	4	18	72.8	95.3	94.9	136.8
P13647	0.0000E+00	16.2712	1	10	5	10	62.3	73.1	68.2	89.7	61.1
P09960	0.0000E+00	9.1653	1	6	6	6	69.2	73.1	70.1	78.4	68.8
P05556	3.0000E-03	3.7594	1	3	3	3	88.4	73.3	94.8	92.3	104.5
Q9ULI3	3.0000E-03	2.3172	1	3	3	4	147.4	73.4	92.6	83.9	98.1
Q6UXB8	0.0000E+00	23.7581	1	8	8	16	49.4	73.5	88.5	81.4	111.9
Q13790	0.0000E+00	11.6564	1	3	3	9	35.4	73.8	57.6	92.1	59.4

Q12860	0.0000E+00	4.9116	1	4	4	4	113.2	74.3	100.8	82	94.2
Q99983	0.0000E+00	11.8765	1	5	5	6	49.5	74.8	89.3	77.1	86.8
Q01459	3.0000E-03	7.7922	1	3	3	3	43.7	75.2	68.4	65.9	71.9
Q7Z7G0	0.0000E+00	7.9070	1	6	6	7	118.6	75.3	81.8	81.7	88.2
P04040	0.0000E+00	21.0626	1	9	9	13	59.7	77	108.2	92.2	129.7
P27797	0.0000E+00	13.9089	1	5	5	5	48.1	77.5	73.6	91.9	90.7
P07900	3.0000E-03	3.6885	1	3	3	3	84.6	79.9	91.7	102.2	105.9
P01598	0.0000E+00	26.8519	1	2	2	2	11.8	79.9	100.6	69.6	82.2
Q6EMK4	0.0000E+00	15.1560	1	6	6	8	71.7	80.6	86.6	80.9	92.5
P33908	3.0000E-03	3.9816	1	3	3	3	72.9	81	91.5	76.3	81.2
P05362	0.0000E+00	17.4812	1	8	8	12	57.8	81.9	80.5	84.7	76.9
P00918	0.0000E+00	30.3846	1	6	6	7	29.2	81.9	128.6	109.5	162.6
P13727	0.0000E+00	27.4775	1	5	5	5	25.2	82.5	71.6	71.9	69
P11279	3.0000E-03	4.0767	1	2	2	3	44.9	83	95.1	99.3	115.7
P27918	0.0000E+00	23.8806	1	10	10	14	51.2	83.2	122.2	106.8	95.6
P13591	0.0000E+00	17.2494	1	11	11	14	94.5	83.9	97.4	90.3	99.1
P00558	5.0000E-03	4.3165	1	2	2	2	44.6	83.9	89.1	86.4	93.4
P23284	6.0000E-03	6.0185	1	1	1	1	23.7	84.1	68.4	82.1	63.5
P15151	0.0000E+00	9.8321	1	4	4	6	45.3	84.3	97.8	98.1	97.5
P06703	0.0000E+00	27.7778	1	2	2	2	10.2	84.3	97	92.1	115.3
P08637	3.0000E-03	7.4803	1	2	2	2	29.1	84.3	101.5	82.1	75.4
P04066	0.0000E+00	6.2232	1	3	3	3	53.7	84.5	95.5	95.7	106.8

P05067	0.0000E+00	7.6623	1	6	6	6	86.9	84.9	75.1	93.2	82.1
P14543	0.0000E+00	8.1796	1	10	10	11	136.3	85.1	89.1	91.9	97.7
Q07954	0.0000E+00	2.1127	1	7	7	7	504.3	85.4	99	107.5	109.6
P01344	0.0000E+00	13.8889	1	2	2	6	20.1	86.5	102.7	90.2	114
O95479	3.0000E-03	4.0455	1	3	3	3	88.8	86.9	77.4	83.4	69.3
Q8NBJ4	0.0000E+00	21.1970	1	7	7	8	45.3	88.9	100	78.5	87.9
P17936	0.0000E+00	48.1100	1	11	11	18	31.7	89.4	125	100.1	135.1
P14780	0.0000E+00	14.1443	1	7	7	7	78.4	89.4	63.2	75.8	60.8
P12830	0.0000E+00	4.9887	1	3	3	4	97.4	89.6	99.7	89.3	86
P10721	0.0000E+00	4.2008	1	4	4	5	109.8	89.8	109.6	73.4	95.7
P49908	0.0000E+00	14.6982	1	7	7	14	43.2	90.7	92.5	100.1	114.6
Q03591	0.0000E+00	20.9091	1	8	1	22	37.6	91	78	94.7	83.1
P55058	0.0000E+00	16.6329	1	6	6	10	54.7	92.2	89.1	120.1	106.6
P07359	0.0000E+00	10.7362	1	6	6	8	71.5	93.6	122.6	113.7	122.2
P18206	0.0000E+00	6.5256	1	8	8	8	123.7	94.2	93.6	105.4	100
P15144	0.0000E+00	8.9969	1	8	8	9	109.5	94.9	86	103.8	82
P04070	0.0000E+00	19.9566	1	8	8	13	52	95.6	104.5	128	107.1
P35908	0.0000E+00	17.5274	1	10	6	12	65.4	97.3	72.4	74.6	63.1
Q9BXR6	0.0000E+00	3.8664	1	2	1	3	64.4	98.2	67.6	94.7	62.3
Q15063	0.0000E+00	12.0813	1	7	7	8	93.3	98.7	95	107.6	106
Q5T619	6.0000E-03	2.1127	1	1	1	11	62.3	99	132.8	133.4	176.7
P09486	0.0000E+00	38.9439	1	10	10	15	34.6	99.3	85.4	98.7	85.7

P01766	0.0000E+00	25.0000	1	2	2	5	13.2	99.6	74.5	73.8	56.1
P40197	0.0000E+00	9.4643	1	5	5	6	60.9	101.2	98.1	119.2	102
P12955	0.0000E+00	8.9249	1	5	5	6	54.5	102.2	123.1	100.4	108.9
P16070	0.0000E+00	5.2561	1	4	4	9	81.5	102.3	127.6	127.3	116.1
P02042	0.0000E+00	93.8776	1	14	7	85	16	102.4	139.2	141.1	176.8
O43505	5.0000E-03	6.0241	1	2	2	2	47.1	103.2	115.4	98.4	118.7
P80188	6.0000E-03	5.5556	1	1	1	2	22.6	103.9	89.6	89.4	55
Q9BTY2	0.0000E+00	7.4946	1	4	4	4	54	106.8	124.8	122.3	152.7
P11021	0.0000E+00	27.8287	1	14	12	15	72.3	107.1	88.7	139.5	104.9
O00187	0.0000E+00	20.1166	1	11	11	18	75.7	109.1	108.6	115.5	113.3
P35443	0.0000E+00	12.6951	1	8	5	9	105.8	110.4	141.6	145.1	174.9
B9A064	0.0000E+00	40.6542	1	6	3	11	23	111.3	108.4	97.9	79.7
P32119	0.0000E+00	37.3737	1	8	8	19	21.9	114.9	174.4	138.2	240.7
P13473	5.0000E-03	2.9268	1	1	1	1	44.9	116.4	126.1	117.9	136.4
P02656	0.0000E+00	37.3737	1	4	4	12	10.8	118.2	94.6	119.4	116.4
P00915	0.0000E+00	36.7816	1	8	8	21	28.9	118.8	206.6	165.6	244.5
P06733	0.0000E+00	19.8157	1	6	6	7	47.1	120.4	132	118.9	130.9
P04406	0.0000E+00	15.8209	1	5	5	6	36	121.3	170.2	168.4	181.7
P14618	6.0000E-03	3.3898	1	2	2	2	57.9	121.7	140.2	153.9	149.1
Q16853	5.0000E-03	3.5387	1	3	3	3	84.6	121.9	157.7	127	158.9
P02746	0.0000E+00	36.3636	1	8	8	27	26.7	122.1	169.6	184.2	164.5
P60709	0.0000E+00	46.1333	1	12	5	30	41.7	122.6	109	137.8	116.7

P05452	0.0000E+00	48.5149	1	8	8	19	22.5	122.8	143.5	152.9	163
Q9NPH3	3.0000E-03	2.8070	1	2	2	3	65.4	124.9	130.7	125.7	138.7
P11226	0.0000E+00	34.6774	1	8	8	16	26.1	126.9	88.9	167.5	109.4
P48740	0.0000E+00	26.7525	1	14	13	25	79.2	127.2	142.3	154.8	166.1
P04180	0.0000E+00	17.0455	1	5	5	8	49.5	127.6	160.5	149.6	148.6
Q96PD5	0.0000E+00	34.0278	1	12	12	36	62.2	127.9	172.8	159.7	217.9
P01857	0.0000E+00	53.0303	1	12	6	32	36.1	128.1	119.4	115.6	99.7
Q9UGM5	0.0000E+00	38.2199	1	12	12	19	42	128.1	143.9	138.2	144.2
Q9UNN8	0.0000E+00	13.4454	1	3	3	3	26.7	128.8	148.2	145.3	131.6
P02751	0.0000E+00	11.0226	1	21	21	27	262.5	129.7	132	155.1	140.6
P01834	0.0000E+00	65.0943	1	4	4	12	11.6	129.9	95	93.8	67.5
Q9NQ79	0.0000E+00	7.4130	1	4	4	5	71.4	130.4	171.7	131.6	143.8
Q9NZP8	0.0000E+00	32.6489	1	10	8	29	53.5	130.6	136.5	140.3	141.8
P05109	0.0000E+00	40.8602	1	5	5	7	10.8	131.7	73.4	110.2	47.4
P22105	0.0000E+00	6.4828	1	20	20	23	457.9	135.3	154.7	147.6	164.1
O14791	0.0000E+00	39.9497	1	13	13	18	43.9	135.8	146.5	138.3	171.2
P01591	0.0000E+00	25.7862	1	4	4	4	18.1	139.4	99.8	98.8	79.9
P00740	0.0000E+00	29.2842	1	11	11	22	51.7	140.1	112.4	141.4	116.8
Q9Y5Y7	0.0000E+00	12.7329	1	4	4	10	35.2	141.4	171.3	192.8	188.5
P0CG05	0.0000E+00	81.1321	1	6	3	13	11.3	141.5	137.9	118.4	121.3
O43866	0.0000E+00	27.0893	1	7	7	10	38.1	142.8	85.2	72.8	64.1
P43251	0.0000E+00	27.9926	1	12	12	20	61.1	143.9	171.3	162.8	173.2

P24821	0.0000E+00	8.9050	1	13	13	14	240.7	143.9	144.8	145.6	157.7
Q9UBR2	3.0000E-03	7.2607	1	2	2	2	33.8	144.5	133.9	121	102.4
P02747	0.0000E+00	34.6939	1	6	6	33	25.8	146.4	189.5	196.5	188.1
O95445	0.0000E+00	42.5532	1	9	9	14	21.2	146.8	153.8	144.8	155.7
P20851	0.0000E+00	51.1905	1	10	10	20	28.3	147.2	138.5	164.6	139.2
P80108	0.0000E+00	18.0952	1	14	14	23	92.3	147.4	176.5	178.9	211.2
Q15485	0.0000E+00	29.3930	1	8	8	17	34	147.8	134	150.3	142.3
P02776	0.0000E+00	35.6436	1	4	4	6	10.8	147.8	137.2	180.3	150.6
Q86VB7	0.0000E+00	15.2249	1	13	13	16	125.4	148.6	157.3	166	129
P05154	0.0000E+00	20.6897	1	8	8	10	45.6	149.3	150.4	145.9	178.1
P02753	0.0000E+00	60.6965	1	8	8	18	23	152.4	165.1	145.2	220.1
Q96KN2	0.0000E+00	37.0809	1	15	15	21	56.7	152.9	246.5	150	165.7
P26927	0.0000E+00	30.5204	1	18	18	27	80.3	153.7	148.9	162.3	163.6
O00391	0.0000E+00	22.7577	1	16	16	20	82.5	154.8	160.5	170.6	163.3
Q92820	0.0000E+00	21.0692	1	6	6	12	35.9	155.2	153	159.6	165.8
Q04756	0.0000E+00	23.8168	1	13	13	26	70.6	165.2	212.1	202.1	218
P07333	0.0000E+00	6.8930	1	6	6	8	107.9	165.8	197.1	209.8	188.7
P14151	0.0000E+00	19.0860	1	6	6	14	42.2	166.5	211.3	162.6	183.6
P80748	5.0000E-03	7.2072	1	1	1	2	11.9	168.5	253.6	140	121.8
P22352	0.0000E+00	32.3009	1	6	6	11	25.5	168.7	161.2	213.8	159.2
P06702	0.0000E+00	66.6667	1	8	8	13	13.2	168.9	97.2	122.4	70.4
P03951	0.0000E+00	34.0800	1	19	19	25	70.1	169.3	133.1	191.6	141.8

P04278	0.0000E+00	46.5174	1	12	12	26	43.8	171.2	205	204.7	241.2
P04075	0.0000E+00	20.3297	1	7	6	8	39.4	172.4	191.8	214.1	215
P02745	0.0000E+00	28.5714	1	5	5	15	26	172.6	225.8	232	211.3
P02671	0.0000E+00	10.9700	1	7	7	9	94.9	179.1	148.3	278.2	146.8
P0C0L4	0.0000E+00	67.7179	1	102	4	862	192.7	180.1	242.7	243.6	296.2
P13645	0.0000E+00	31.1644	1	18	14	27	58.8	186.6	129.9	157.7	127.6
P35527	0.0000E+00	44.1413	1	20	19	45	62	189.7	199.7	286.4	186.7
P80511	0.0000E+00	9.7826	2	3	2	1	10.6	192.6	60.3	82.1	65.1
Q13085	6.0000E-03	0.2984	1	1	1	4	265.4	196.6	170.6	258.1	172.7
Q9UK55	0.0000E+00	36.7117	1	14	14	18	50.7	197.1	175.6	173.8	144.9
P55056	0.0000E+00	39.3701	1	6	6	12	14.5	197.6	171.7	241.8	178.2
P04264	0.0000E+00	46.8944	1	24	23	40	66	200.9	207.3	296.6	176.8
P62328	0.0000E+00	40.9091	1	2	1	5	5.1	201.4	187.7	247.3	198.5
P02655	0.0000E+00	49.5050	1	4	4	12	11.3	202.1	122.6	160.7	165.5
P13796	0.0000E+00	36.6826	1	19	19	24	70.2	208.4	173.4	220	175.3
Q15582	0.0000E+00	24.3045	1	14	14	22	74.6	213.5	177.5	245.7	217.4
P01876	0.0000E+00	37.3938	1	9	9	12	37.6	216.8	167.9	181.1	119.1
Q8TAQ9	6.0000E-03	1.9608	1	1	1	4	40.5	218	185	252.1	187.3
P15169	0.0000E+00	41.9214	1	12	12	38	52.3	223.8	213.1	221.6	232.9
P15311	6.0000E-03	2.7304	1	2	2	2	69.4	224.3	265.5	278.9	270.6
P08571	0.0000E+00	41.3333	1	14	14	16	40.1	230.1	258.5	248.7	238.8
P02775	0.0000E+00	44.5313	1	7	7	35	13.9	231.2	193.4	253.6	227.2

Q16610	0.0000E+00	51.6667	1	20	20	30	60.6	236.9	247.4	204.1	223.4
P27169	0.0000E+00	43.9437	1	11	9	28	39.7	239.4	268.3	265.7	269.3
P00742	0.0000E+00	33.6066	1	15	15	42	54.7	240.1	249.7	295.4	280.5
P06276	0.0000E+00	33.2226	1	18	18	38	68.4	241.7	344.9	268.4	300.3
P61626	0.0000E+00	18.9189	1	3	3	5	16.5	246.3	221.2	217.9	240.2
O75636	0.0000E+00	36.1204	1	10	10	32	32.9	253.5	251.9	253.5	240.9
Q92954	0.0000E+00	12.7493	1	17	17	24	151	254.3	283.6	325.3	299.1
P05090	0.0000E+00	22.7513	1	5	5	21	21.3	256.6	232.4	269.7	242.9
P35542	0.0000E+00	45.3846	1	7	7	28	14.7	258.6	271.4	286.3	306.1
P10909	0.0000E+00	29.3987	1	13	13	49	52.5	262.3	248.9	389.7	311.4
P01871	0.0000E+00	48.0088	1	18	7	52	49.3	268.3	172.1	150.7	135.6
P35858	0.0000E+00	42.4793	1	20	20	51	66	270.1	442.2	347.4	490.4
P0C0L5	0.0000E+00	68.0619	1	103	5	896	192.6	282.3	390.7	344	410.5
P12259	0.0000E+00	16.0971	1	34	34	47	251.5	282.8	275.9	312.8	295.9
Q14520	0.0000E+00	35.1786	1	14	14	39	62.6	287.1	249	300.3	281.9
P54108	5.0000E-03	6.5306	1	2	2	3	27.6	296.3	330.5	325.4	276.8
P02652	0.0000E+00	69.0000	1	8	8	79	11.2	302.1	468.7	304.8	476.9
P02654	0.0000E+00	32.5301	1	4	4	14	9.3	306.8	287.6	309.2	324.6
P29622	0.0000E+00	50.5855	1	17	17	39	48.5	308	351.3	396	464.3
P00739	0.0000E+00	68.6782	1	22	8	483	39	312	220.6	274.2	186.4
P07225	0.0000E+00	35.7988	1	19	19	58	75.1	319.6	275.1	361.6	283
P0DJI8	0.0000E+00	72.1311	1	10	4	86	13.5	319.7	293.7	225	178.9

P68871	0.0000E+00	95.2381	1	16	9	157	16	326.8	490.9	474.8	651.7
P02741	0.0000E+00	24.5536	1	6	6	27	25	328.2	164	336.4	172.2
P07360	0.0000E+00	68.8119	1	10	10	66	22.3	335	320.5	335.9	316.5
P07996	0.0000E+00	35.8120	1	32	32	58	129.3	336	301.3	378	389.3
P18428	0.0000E+00	25.1559	1	9	9	13	53.4	337	230.4	352.1	193.6
P19320	0.0000E+00	33.0176	1	20	20	28	81.2	342.2	293.8	338.3	338.9
P02766	0.0000E+00	73.4694	1	12	12	125	15.9	355	460.4	381.8	510
Q96IY4	0.0000E+00	34.9882	1	12	12	27	48.4	355.7	271.8	403	302.1
P51884	0.0000E+00	40.8284	1	11	11	55	38.4	356.5	462.3	404.5	464.6
O75882	0.0000E+00	26.3821	1	31	31	93	158.4	393.4	532.7	487.5	563.9
P36955	0.0000E+00	37.3206	1	14	14	52	46.3	394.7	362.6	351	375.4
P06681	0.0000E+00	38.4309	1	26	26	86	83.2	397.2	429	445.3	429.5
Q08380	0.0000E+00	41.3675	1	21	21	95	65.3	411.2	620.4	442.9	497
P02743	0.0000E+00	34.0807	1	9	9	62	25.4	427.4	436.7	466.8	397.2
P07358	0.0000E+00	49.9154	1	24	24	83	67	434.9	433.7	438.4	448.1
P03952	0.0000E+00	54.2320	1	31	31	82	71.3	464.6	508	526.3	534.5
P06396	0.0000E+00	36.8286	1	26	26	74	85.6	469.2	534.1	489.6	582.1
P05546	0.0000E+00	55.1102	1	22	22	79	57	488.8	502.1	643.8	615.5
P09871	0.0000E+00	45.3488	1	23	22	89	76.6	489.6	472.9	497.1	483.1
P02749	0.0000E+00	57.3913	1	14	14	89	38.3	491.1	399.7	422.1	395
P08697	0.0000E+00	53.5642	1	22	22	80	54.5	498.6	506.2	507.5	529.9
P01019	0.0000E+00	40.4124	1	15	15	77	53.1	501.9	434.8	550	492.9

P07357	0.0000E+00	45.8904	1	21	21	85	65.1	503	472.2	508.3	485.6
P05543	0.0000E+00	40.0000	1	17	17	47	46.3	505.5	418.2	552.1	601.2
P04196	0.0000E+00	35.6190	1	17	17	112	59.5	515.2	654.5	580	499.6
P02787	0.0000E+00	64.6132	1	43	43	106	77	520.7	692.5	616.6	695.2
P00734	0.0000E+00	59.9678	1	27	27	169	70	522.4	511.6	698.5	602.9
P08185	0.0000E+00	50.8642	1	14	14	93	45.1	541.8	616.5	605.5	645.8
P02748	0.0000E+00	46.3327	1	23	23	107	63.1	558	478.2	523.5	438.7
P00736	0.0000E+00	51.0638	1	27	25	100	80.1	573.8	536.8	619.9	583.4
P05156	0.0000E+00	45.1115	1	23	23	119	65.7	595.7	544.5	612.5	553.9
P02760	0.0000E+00	43.7500	1	13	13	127	39	604.3	562.4	519.8	576.1
P02649	0.0000E+00	71.6088	1	26	26	121	36.1	604.8	589.3	908	628.3
P04004	0.0000E+00	43.0962	1	16	16	135	54.3	621.5	618	629.5	719.7
P22792	0.0000E+00	45.5046	1	17	17	69	60.5	622.8	616.4	694.8	743
P69905	0.0000E+00	95.0704	1	12	12	118	15.2	641.1	892.5	905.5	1095.5
P10643	0.0000E+00	53.0249	1	34	33	136	93.5	642	573.7	680.7	710.2
P02750	0.0000E+00	50.4323	1	13	13	78	38.2	642.9	469	560.7	401.3
P13671	0.0000E+00	49.1435	1	40	40	114	104.7	667.3	718.1	781.1	749
P43652	0.0000E+00	60.6010	1	34	34	120	69	703.8	991.9	848.5	1078.4
P04275	0.0000E+00	32.5986	1	74	74	107	309.1	725.1	617	678.3	639.2
Q06033	0.0000E+00	33.0337	1	25	25	119	99.8	767.6	558.1	718.6	562.9
P19652	0.0000E+00	48.2587	1	11	8	193	23.6	770.8	399.5	718.3	525.4
P02765	0.0000E+00	50.6812	1	12	12	190	39.3	777.2	1055.6	761	1017.1

P25311	0.0000E+00	55.7047	1	18	18	129	34.2	859.1	744.2	828.7	711.1
P06727	0.0000E+00	75.5051	1	33	33	147	45.4	859.3	787.5	863.7	1009.7
P01008	0.0000E+00	69.8276	1	34	34	180	52.6	880.9	827.7	883.8	1012.4
P04003	0.0000E+00	67.8392	1	32	32	220	67	909.4	797.9	957.9	788.6
P08603	0.0000E+00	45.7352	1	52	47	234	139	1007	897.6	1230	850.4
P04217	0.0000E+00	61.6162	1	20	20	242	54.2	1072.3	1114.8	1226.2	1146.2
P19827	0.0000E+00	43.0296	1	29	29	270	101.3	1201.1	1304.6	1217.9	1364.2
P00747	0.0000E+00	64.6914	1	49	49	270	90.5	1221.5	1185.7	1411.5	1397.9
P01042	0.0000E+00	43.1677	1	34	34	242	71.9	1297.8	1192.2	1317.3	1202.4
P02768	0.0000E+00	80.4598	1	52	52	281	69.3	1324	1830.5	1398.6	1760.3
P19823	0.0000E+00	52.6427	1	41	41	307	106.4	1328	1540.5	1414.8	1635.5
Q14624	0.0000E+00	55.5914	1	45	45	275	103.3	1592.3	1302.3	1653.4	1342.6
P01031	0.0000E+00	56.5036	1	81	81	295	188.2	1949	1704.2	2344.6	1753.5
P05155	0.0000E+00	42.6000	1	23	23	336	55.1	1977.2	1850.8	2124.7	2058.3
P02763	0.0000E+00	44.7761	1	10	7	394	23.5	1985.1	1090.8	1843.6	1054.8
P02647	0.0000E+00	81.2734	1	29	29	394	30.8	2186.9	2700.9	2021.3	2545.5
P00738	0.0000E+00	67.4877	1	30	16	883	45.2	2459.5	1605.7	2949.7	1757.5
P00751	0.0000E+00	61.5183	1	44	44	438	85.5	2524.2	2462.1	2526.1	2343.2
P02774	0.0000E+00	84.5992	1	39	39	623	52.9	2540.6	2942.1	2900.8	3048.1
P02790	0.0000E+00	65.1515	1	27	27	563	51.6	2819	2829.7	2969.1	2954
P00450	0.0000E+00	71.3615	1	60	60	682	122.1	3141.7	2827.9	3325.3	2821.6
P01011	0.0000E+00	60.0473	1	26	26	664	47.6	3360.4	2267.6	3457	2375.5

P01023	0.0000E+00	72.9308	1	87	77	1226	163.2	4758.6	5197	4866	5492
P04114	0.0000E+00	62.0644	1	266	266	1117	515.3	6250	5361.2	7635.4	6046.7
P01009	0.0000E+00	72.0096	1	41	40	1097	46.7	7345.9	3094.8	6742.2	3461.8
P01024	0.0000E+00	85.8088	1	144	144	2515	187	12497.6	10821.6	12978.5	11242.9
